# Design, Development, Evaluation, and In Vivo Performance of Buccal Films Embedded with Paliperidone-Loaded Nanostructured Lipid Carriers

**DOI:** 10.3390/pharmaceutics15112530

**Published:** 2023-10-25

**Authors:** Fahad Mohammed AlMulhim, Anroop B. Nair, Bandar Aldhubiab, Hiral Shah, Jigar Shah, Vivek Mewada, Nagaraja Sreeharsha, Shery Jacob

**Affiliations:** 1 Department of Pharmaceutical Sciences, College of Clinical Pharmacy, King Faisal University, Al-Ahsa 31982, Saudi Arabia; 221401816@student.kfu.edu.sa (F.M.A.); baldhubiab@kfu.edu.sa (B.A.); sharsha@kfu.edu.sa (N.S.); 2 Department of Pharmacy Services, Johns Hopkins Aramco Health Care (JHAH), Dharan 34464, Saudi Arabia; 3 Department of Pharmaceutics, Parul College of Pharmacy and Research, Parul University, Ahmedabad 380058, India; vyashiral@yahoo.co.in; 4 Department of Pharmaceutics, Institute of Pharmacy, Nirma University, Ahmedabad 382481, India; jigsh12@gmail.com (J.S.); vmewada91@gmail.com (V.M.); 5 Department of Pharmaceutics, Vidya Siri College of Pharmacy, Off Sarjapura Road, Bangalore 560035, India; 6 Department of Pharmaceutical Sciences, College of Pharmacy, Gulf Medical University, Ajman 4184, United Arab Emirates; sheryjacob6876@gmail.com

**Keywords:** buccal film, paliperidone, nanostructured lipid carriers, Box-Behnken design, pharmacokinetics

## Abstract

The therapeutic effectiveness of paliperidone in the treatment of schizophrenia has been limited by its poor oral bioavailability; hence, an alternative route could be appropriate. This study investigates the feasibility of developing a buccal film impregnated with paliperidone-loaded nanostructured lipid carriers (NLCs) and assesses the potential to enhance its bioavailability. Box–Behnken-based design optimization of NLCs was performed by examining the particles’ physical characteristics. The polymeric film was used to load optimized NLCs, which were then assessed for their pharmaceutical properties, permeability, and pharmacokinetics. The optimization outcomes indicated that selected formulation variables had a considerable (*p* < 0.05) impact on responses such as particle size, entrapment efficiency, and % drug release. Desired characteristics such as a negative charge, higher entrapment efficiency, and nanoparticles with ideal size distribution were shown by optimized NLC dispersions. The developed film demonstrated excellent physico-mechanical properties, appropriate texture, good drug excipient compatibility (chemically stable formulation), and amorphous drug nature. A sustained Weibull model drug release (*p* < 0.0005) and superior flux (~5-fold higher, *p* < 0.005) were seen in NLC-loaded film compared to plain-drug-loaded film. The pharmacokinetics profile in rabbits supports the goal of buccal therapy as evidenced by significantly higher AUC_0–12_ (*p* < 0.0001) and greater relative bioavailability (236%) than the control. These results support the conclusion that paliperidone-loaded NLC buccal film has the potential to be an alternate therapy for its effective administration in the treatment of schizophrenia.

## 1. Introduction

Schizophrenia is a severe and debilitating mental illness that often manifests a range of symptoms and generally affects the quality of life of the working-age population. The exact cause of schizophrenia is still unknown, but it is believed to be a combination of genetic, environmental, and neurobiological factors. Cognitive, positive, and negative symptoms among individuals with schizophrenia can have an impact on many elements of their daily life [1]. This disabling health condition is typically treated with medication and psychotherapy. Several drugs are available for early and maintenance therapy with the aim of controlling symptoms, despite the fact that there is no known cure for schizophrenia [2]. Antipsychotic medications can help to alleviate the positive symptoms of schizophrenia, such as hallucinations and delusions. Atypical new-generation antipsychotics are the preferred medications for the initial management of schizophrenia, due to their higher tolerability profile and greater range of clinical action [2,3]. In addition, these second-generation antipsychotics are capable of producing lesser extrapyramidal symptoms at therapeutic levels and have a better patient-reported life quality [4].

Paliperidone, a long-acting second-generation antipsychotic, has been shown to reduce acute schizophrenia symptoms and provide clinical benefits in both acute and maintenance therapy in adults [5]. Typically, paliperidone (9-OH-risperidone) is a pharmacologically active metabolite of risperidone with a distinct pharmacokinetics profile from the parent compound. The proposed therapeutic activity in the treatment of schizophrenia is due to its high affinity and antagonistic activity towards dopamine (D2) and serotonin (5-HT2A) type 2 receptors [6]. The drug is commercially available as extended-release oral tablets and an intramuscular suspension with similar safety profiles and is well tolerated [5]. However, the oral bioavailability of paliperidone is very low (~28%), owing to its extremely poor aqueous solubility [7,8]. Hence, employing formulation techniques to enhance solubility and/or finding an alternative route of administration to improve bioavailability is necessary for its effective therapeutic use [9]. Numerous approaches have been explored through extensive research to enhance the effectiveness of paliperidone. Formulation techniques such as cocrystallization [10], lipid nanoconstructs [8], and ion-exchange resin complexes [11] were attempted. Similarly, transdermal and nose-to-brain routes were also assessed [4,9,12,13]. 

The buccal mucosal membrane is an attractive site for delivering drugs owing to its unique characteristics such as high blood flow, high permeability, low enzymatic activity, and less sensitivity. The buccal route has various advantages over other routes of administration which include avoiding acid hydrolysis, bypassing the first-pass metabolism, enhancing bioavailability, providing rapid onset of action, being noninvasive, being effective for local or systemic action, and being easy to administer or remove in the case of adverse effects [14]. Mucoadhesive drug delivery systems are capable of adhering to the highly vascularized mucus membrane, providing faster drug absorption through the jugular vein, and transporting the drug to the heart directly [15]. Considering the significance of buccal therapy, the US FDA has approved several therapeutic actives (buprenorphine, fentanyl, naloxone, lidocaine, etc.) that have been commercially successful [16]. Among buccal formulations, biocompatible mucoadhesive films are a popular dosage form owing to their versatility, adaptability, ease of scaleup, light weight, physical flexibility, customized size, and high patient compliance [17]. In addition, the buccal films are capable of providing rapid onset and extended duration of action, which are ideal for the management of schizophrenia episodes. The characteristics of paliperidone, including its molecular weight (426.48 Da), lipophilicity (log p =1.8), melting point (179.8 °C) and dose (3–12 mg/day), support its prospects for buccal therapy. However, this drug belongs to BCS class II (practically insoluble in water) and hence requires solubility enhancement to improve bioavailability.

Nano-drug carriers have the potential to address numerous biopharmaceutical obstacles that are associated with the delivery of drugs through the buccal route. The potential of nanoparticles incorporated in buccal film for drug delivery has recently attracted attention [15,18,19]. In this regard, nanostructured lipid carriers (NLCs) have been extensively studied as versatile and efficient vesicles that can enhance the solubility, dissolution rate, bioavailability, and therapeutic efficacy of inadequately soluble drugs across various drug delivery systems [20]. Several studies have reported the ability of NLCs to increase the solubility and diffusion coefficient of drugs across the mucosal epithelial layers and the stability of drugs against degradation in the buccal environment [21,22,23,24]. Compared to solid lipid nanoparticles, the utilization of both solid and liquid lipids in NLCs enables higher drug loading, minimal drug loss during storage, and sustained drug release [25,26]. 

Meanwhile, the fabrication of a buccal film with desirable properties requires polymers with film-forming and mucoadhesive properties. In general, the films prepared using two different polymers exhibit improved mucoadhesive and physical properties due to the combination of their constituent polymers. From this perspective, hydroxypropyl methylcellulose (HPMC) possesses good film-forming properties, high water-absorbing capacity, rapid swelling, and mucoadhesive properties, making it a popular choice for formulating buccal films [17]. Similarly, polycarbophil (PC), a high-molecular-weight acrylic acid polymer, has good bioadhesive properties and can increase buccal retention by entangling within the mucus layer [27]. Given that both HPMC and PC possess favorable biological and mucoadhesive properties, these combinations were explored in bioadhesive drug delivery systems [27,28]. Therefore, this study aimed to develop and optimize paliperidone-loaded NLCs embedded in a buccal mucoadhesive film and preliminarily evaluate their potential as an alternative therapy for schizophrenia. Solid (glycerol monostearate) and liquid (oleic acid) lipids were selected according to the preliminary solubility study results, and the NLCs were formulated using the heat homogenization process. The Box–Behnken experimental design was utilized to develop paliperidone-loaded NLCs by evaluating the influence of formulation components (lipid content, surfactant concentration, and ultrasonication time) on particle properties. The optimized NLCs with suitable characteristics were successfully impregnated in a film containing HPMC and PC and evaluated for film characteristics, drug release, permeation, and absorption in rabbits.

## 2. Materials and Methods

### 2.1. Chemicals

A complimentary sample of paliperidone (MW, 426.49 Da) was acquired from Emcure Pharmaceuticals, Gandhinagar, India. Tween 80 and glyceryl monostearate were purchased from CDH Pvt. Ltd., Mumbai, India. Labrafil^®^ M 2125 CS, Labrafil^®^ M 1944 CS, and Labrafac^®^ CC of USPNF/EP quality were purchased commercially (Gattefosse, Saint-Priest, France). Hydroxypropyl methylcellulose (HPMC) K4M and polycarbophil (PC) were obtained from Chemidyes, Ahmedabad, India. Oleic acid was procured from Sigma Aldrich, Munich, Germany. Methanol and acetonitrile used in high-performance liquid chromatography (HPLC) analysis were obtained from Fisher Scientific (Loughborough, UK).

### 2.2. Quantification of Paliperidone

Estimation of paliperidone in samples was carried out using Shimadzu Prominence HPLC (Tokyo, Japan) system equipped with a C18 Inertsil ODS 3V (4.6 × 250 mm × 5 µm) column coupled to a UV–Vis detector. A mobile phase comprising methanol–acetonitrile (40:60 *v*/*v*) was utilized for chromatographic separation of paliperidone. The separation was performed at a flow rate of 1 mL/min and an injection volume of 25 µL. The isocratic elution was performed at 25 °C and was monitored using a UV detector at 235 nm [29], and the retention time was 3.24 min. Analytical method validation studies were performed to assess sensitivity, selectivity, linearity, precision, accuracy, and protein precipitation. The linearity of the PA was in the concentration range of 10–200 ng/mL with a higher regression coefficient (r^2^ = 0.9983). The method was validated as per ICH guidelines in simulated salivary fluid (SSF, pH 6.8). The limit of quantification (LOQ) was determined as 7.50 ng/mL, while the limit of detection (LOD) was found to be 5.72 ng/mL. The coefficient of variation for the method ranged from 1.16% to 2.84%. Additionally, the recovery of PA from plasma was determined to be 97.35% ± 2.3%.

### 2.3. NLC Formulation Preliminary Studies

The solubility of paliperidone in solid lipids (glycerol monostearate, Precirol ATO 5, Compritol 888 ATO, Dynasan 114, and stearic acid) was assessed by the method described earlier [30]. In brief, 2 mg of paliperidone was taken in 5 mL glass vials, and the solid lipid was added in increments. The vials were kept in a water bath (80 °C), and the amount of lipid required to solubilize the drug completely was noted.

The conventional equilibrium solubility method described in the literature [30] was employed to evaluate the solubility of paliperidone in liquid lipids. Briefly, 1 g of selected liquid lipids (oleic acid, Labrafil^®^ M 2125 CS, Labrafil^®^ M 1944 CS, and Labrafac^®^ CC) was taken in glass vials, and an excess amount of drug was added. The vial was placed in a shaker water bath at room temperature for 24 h and then equilibrated by keeping it at rest for 1 h. The mixture was centrifuged (7885× *g* for 15 min), and the upper layer (200 µL) was mixed with methanol (2 mL) to extract paliperidone. The methanolic extract was diluted with methanol, filtered using a membrane filter with a 0.22 µm pore size, and analyzed by HPLC.

### 2.4. Preparation of NLCs

NLCs were prepared with a hot homogenization method as previously mentioned [31]. Briefly, aqueous and oil phases were prepared individually by weighing the required amount of formulation components. The aqueous phase was constituted of Tween 80 and water, while the oil phase contained the drug (paliperidone), oleic acid, and glycerol monostearate. The temperature of both phases was raised to the predetermined level by heating each phase independently to 80 °C. Then, the aqueous phase was dispersed into the oil phase using an Ultra-Turrax^®^ T25 digital high-shear homogenizer (IKA^®^, Staufen, Germany) at 8000 rpm for 3 min. The primary emulsion formed was size-reduced with the help of a probe sonicator (Sonics and Materials Inc, Newtown, CT, USA) for 15 min (3 cycles of 5 min) at 40% amplitude. The NLC dispersion was subsequently cooled to room temperature. The NLC dispersion (10 mL) was mixed with 5% mannitol (cryoprotectant) and stored for freezing (−80 °C for 12 h), followed by primary (−20 °C for 12 h) and secondary drying (25 °C for 2 h) to obtain a dry and free-flowing powder [32]. 

### 2.5. Optimization of NLCs by Box–Behnken Method

Using Design Expert software (Version 13), a three-factor, three-level Box-Behnken design was employed to investigate the quadratic response surfaces and create second-order polynomial models [33]. The design matrix included five duplicated center points and 17 experimental runs; the actual and coded values for the individual experimental runs are outlined in Table 1 and Table S1. The polynomial equation for this model is written as follows:

$$Y=\beta_{0}+\beta_{1}X_{1}+\beta_{2}X_{2}+\beta_{3}X_{3}+\beta_{12}X_{1}X_{2}+\beta_{13}X_{1}X_{3}+\beta_{23}X_{2}X_{3}+\beta_{11}X_{1}^{2}+\beta_{22}X_{2}^{2}+\beta_{33}X_{3}^{2}$$

where Y is the measured response related to each factor level combination; β_0_ is constant; β_1_, β_2_, and β_3_ are linear coefficients; β_12_, β_13_, and β_23_ are interaction coefficients between the three factors; β_11_, β_22_, and β_33_ are quadratic coefficients of the observed experimental values; and X_1_, X_2_, and X_3_ are the levels of independent variables that have been mentioned.

### 2.6. Characterization of NLCs

#### 2.6.1. Particle Characterization

The prepared vesicle’s particle size, size distribution, polydispersity index, and zeta potential were analyzed using Zetasizer (Horiba SZ-100, Kyoto, Japan) in SSF at 25 °C. The individual samples of each formulation were placed in disposable polystyrene cuvettes and oriented toward the laser light beam. A detector was positioned at a perpendicular angle to measure the scattered light signal, and the particle size was determined using the physical properties of the scattered light. To measure the zeta potential, the samples were dispersed in SSF, and their electrophoretic mobility values were ascertained at 25 °C using the Horiba Zetasizer. Each measurement was performed in triplicate to ensure accuracy and consistency.

#### 2.6.2. Entrapment Efficiency (EE) and Drug Loading

The drug level in both the aqueous phase and inside the carrier was measured to assess the EE and loading capacity of paliperidone in prepared NLCs [34]. The total amount of drug inside the carriers was determined after dissolving 2 mL (~200 mg NLC) of dispersion in anhydrous methanol (20 mL). Similarly, the amount of free drug was estimated using 2 mL of samples, centrifuged (3080× *g* for 30 min) using a millipore Amicon filter (MWCO 10 kDa, Darmstadt, Germany), and the filtrate solution obtained was measured. The EE was determined using the following formula:

EE%=[(Total amount of drug−amount of free drug)/Total amount of drug]×100

Similarly, the drug loading was determined using the following formula:

%DL=[(Total amount of drug−amount of free drug)/Total amount of NLC]×100

#### 2.6.3. Drug Release

The dialysis bag method was used for the evaluation of paliperidone release from formulated NLCs. A dialysis membrane with 12–14 kDa MWCO (Spectra/por^®^ Spectrum Laboratories Inc. Rancho Dominguez, Berkeley, CA, USA) was utilized for the study and was previously soaked in SSF (pH 6.8) overnight. Samples of NLCs (containing 2 mg of paliperidone) were mixed in SSF and kept inside the membrane and immersed in a beaker containing 100 mL of receiver fluid (SSF with 0.5% Tween 80; pH 6.8; solubility 0.82 ± 0.11 mg/mL) to maintain the sink condition [35]. The complete apparatus was placed on a water bath adjusted to maintain a temperature of 37 ± 0.5 °C, and the receptor medium was agitated at 50 rpm. Samples of the receptor medium (1 mL) were withdrawn at intervals of up to 6 h and replaced with an equivalent volume of fresh medium. The samples were then diluted and analyzed for drug content using the HPLC method described earlier. Various mathematical models were used to analyze the data and determine the correlation coefficient (r^2^) and release kinetics.

### 2.7. Morphology

#### 2.7.1. Optical Microscopy

The appearance of the prepared NLCs was observed using optical microscopy (X21iLED, Olympus, Germany). Briefly, the formulation was diluted with water (1:2), kept over a glass slide (76 × 26 × 1 mm), and observed under a microscope with a 40× objective and 10× ocular lens. 

#### 2.7.2. Transmission Electron Microscopy (TEM)

The surface morphology and vesicle size/shape of prepared NLCs were observed using TEM (JEM 2100 TEM HR LaB6 Version, JEOL, Tokyo, Japan) operating at 100 kV. A portion of the formulation was taken and mixed with distilled water, and a small amount of the resulting dispersion was deposited onto carbon-coated copper grids, which were then air-dried at room temperature (25 ± 0.5 °C). The grid was fixed to the device, and images at a magnification of 2000× were taken. 

### 2.8. Preparation of Buccal Mucoadhesive Film

The film casting method was used to formulate HPMC/PC mix films, as previously described [23]. In brief, a clear aqueous solution of HPMC (2% *w*/*v*) was prepared separately and added to PC dispersion (0.5% *w*/*v*) progressively with constant stirring for 30 min to achieve homogeneity. The mixture was supplemented with glycerol (1%), a plasticizer, and stirred for an additional 15 min. The NLC dispersion (25 mL) was added to the HPMC/PC composite solution (75 mL) and the combination was well blended for 15 min. The produced dispersion was transferred onto a glass plate (25 cm^2^) and placed in a warm air oven to dry for 10 h at 50 °C. The NLC-loaded film was affixed to a backing membrane composed of 5% (*w*/*v*) ethyl cellulose and 2% (*v*/*v*) dibutyl phthalate using a polyvinylpyrrolidone adhesive polymer (5% *w*/*v*). To test different film qualities, HPMC/PC blend films with paliperidone were formulated and utilized as a control.

### 2.9. Characterization of Buccal Mucoadhesive Film 

The thickness of prepared patches was measured at multiple locations using a vernier caliper (Mitutoyo, Kawasaki, Japan). The pH of the film was determined by placing the electrode on the surface of the film, which was previously soaked in distilled water for an hour, and the surface was wiped with cotton. The folding endurance of the film was determined by repeatedly folding it along the same axis (4 cm^2^). The amount of paliperidone in films was tested by taking film (1 cm^2^) from various locations and soaking it overnight in the methanol–water mixture in a shaker water bath. The drug in the solvent was assayed by HPLC. 

The swelling index or % hydration of drug-loaded and NLC-loaded films was tested. A section of the weighed film (W_1_, 1 cm × 1 cm) was placed on a metallic wire mesh and immersed in 10 mL of SSF (pH 6.8) set at 37 ± 1 °C. The weight (W_2_) of films at different time intervals was checked, and % hydration was calculated as follows [36]:

$$\%hydration=W_{2}-W_{1}/W_{1}\times 100$$

### 2.10. Tensile Strength 

The tensile strength of the optimized NLC-loaded film and placebo was measured using a texture analyzer (QTS-25, Brookfield Engineering Labs, Middleboro, MA, USA) calibrated with a 5 kg load cell [37]. The films were cut into 7 cm length and 2 cm width strips and fixed between two tensile grips positioned 3 cm apart. The films were subjected to a tensile test at a speed of 20 mm/s until they reached their breaking point. The tensile strength was measured according to the formula described in the literature [37].

### 2.11. Mucoadhesive Strength

The mucoadhesive strength of the control film (PA-Film) and the optimized PA-NLC film was evaluated using a texture analyzer used in tensile strength measurement and rabbit buccal mucosa as the substrate. Briefly, the buccal membrane, which had been previously moistened with SSF, was securely fixed to the stationary platform. A film measuring 1 cm^2^ was then affixed to the probe of the analyzer. Gradually, the movable probe was lowered until it contacted the mucus membrane and remained in place for 1 min. The measurement of mucoadhesive strength followed the parameters outlined in the literature [38]. 

### 2.12. Degree of Crystallinity

Thermal analysis of paliperidone, glycerol monostearate, physical mixture, NLC, control film (plain-drug-loaded film), and optimized NLC-loaded film was performed employing a differential scanning calorimetry (DSC) instrument (DSC 7020, Hitachi, Tokyo, Japan). The device was calibrated using pure indium as the standard. Samples (5 mg) were precisely weighed and placed in aluminum crimped pans, which were then sealed non-hermetically. An empty pan was used as a reference standard during the study. Thermograms of the samples were recorded between 30–300 °C at a heating rate of 10 °C/min after being brought to equilibration at 25 °C for 5 min. The drug, polymer, and film were subjected to thermal scanning in a nitrogen atmosphere between 30 °C and 300 °C at a heating rate of 10 °C/min.

### 2.13. Spectral Analysis 

Spectral characteristics of paliperidone, glycerol monostearate, the physical mixture, NLCs, the control (plain-drug-loaded film), and the optimized NLC-loaded film were documented using an FTIR spectrometer (FT/IR-6100, Jasco, Tokyo, Japan). A small amount of the samples was combined with KBr powder at a ratio of 1:5 and ground using a mortar and pestle, and discs were made using a hydraulic press. The discs were then placed into a stainless-steel sample holder, and IR spectra between 400 and 4000 cm^−1^ were captured. 

### 2.14. Paliperidone Release from Films

The release of paliperidone from NLC-loaded and control films was assessed using a USP Type II device (Electrolab TDC 50, Mumbai, India), [39]. The dissolving medium (900 mL) had SSF with 0.5% Tween 80 to maintain the sink condition. The prepared film (1 cm × 1 cm containing 2 mg of paliperidone) was pasted to a glass slide such that the drug could be released in the direction of the release medium and placed in the bottom of the jar. The assembly was maintained at a temperature of 37 ± 0.5 °C, and the speed of the paddle was adjusted to 50 rpm. The collected samples were passed through a syringe membrane filter (0.2 µm, Millipore, Bedford, MA, USA) and analyzed using HPLC. Mathematical release kinetics models, including zero-order, first-order, Higuchi, Korsmeyer–Peppas, Weibull, and Hixon–Crowell, were applied to the drug release data. KinetDS 3.0 software was utilized to determine the drug release pattern of the formulated buccal films.

### 2.15. Drug Permeation

Isolated fresh rabbit buccal mucosae were used in paliperidone permeation from NLC-loaded and control films. The rabbit buccal mucosa membrane was placed between the donor and receptor chambers of a Franz-type diffusion setup. The resistance of buccal epithelium was measured according to the method described earlier [40], and the membrane that had a resistance of >3 kΩ·cm^2^ was used. Prepared films with the specified area (1 cm × 1 cm) containing 2 mg of paliperidone were placed between two chambers. The permeation medium was SSF adjusted to a pH of 6.8 (10 mL), temperature of 37 ± 0.5 °C, and stirring speed of 50 rpm. An adequate amount of samples was taken from the receiver, centrifuged (10,351× *g*, R-83; Remi, Mumbai, India) for 10 min, and assayed by HPLC. The flux and permeability coefficient were calculated based on the literature [41].

### 2.16. Pharmacokinetics in Rabbits

Twelve male rabbits weighing 2.5–3 kg were housed in an animal facility for one week before the initiation of the experiment. Guidelines mentioned in the university animal ethical approval (KFU-REC-2021-NOV-EA000158, dated 9 November 2021) were strictly followed for all experimental procedures. Rabbits were anesthetized (2.5–3 h) using recommended medium anesthesia: ketamine (40 mg/kg) and xylazine (5 mg/kg) by intramuscular route. In one group of animals (*n* = 6), the NLC-loaded film (1 cm^2^ size, containing 2 mg of paliperidone) was cut into two halves and applied bilaterally to the inner cheeks of rabbits, while in another group (*n* = 6), an equivalent dose of paliperidone as an oral suspension (2 mg/mL) was administered. The dose of paliperidone in rabbits was calculated based on the human dose of 12 mg, applying a conversion factor as recommended [42]. Blood samples (500 µL) were collected (1, 2, 3, 4, 6, 8, and 12 h) from the marginal ear vein and mixed with an equal amount of acetonitrile (to precipitate plasma proteins), vortexed (5 min), and centrifuged (5976× *g* for 5 min at 4 °C). The drug containing the top layer was injected into the HPLC. 

### 2.17. Data Assessment

Statistical significance was estimated using Graph Pad Prism^®^ 6 (GraphPad Software Inc., San Diego, CA, USA). The criterion for statistical significance was *p* < 0.05.

## 3. Results and Discussion

### 3.1. Selection of Lipids

Adequate drug solubility in the lipid system is an essential parameter in developing an efficient lipid-based formulation because it is directly linked to the dose that can be delivered [43]. In the case of NLCs, the lipid with the highest drug solubility results in greater drug loading while keeping the drug solubilized inside the carrier. On the other hand, when choosing lipids, care should be taken to consider their physicochemical properties, stability, biocompatibility, toxicity, etc. [24]. Thus, the initial part of the study included screening and identification of suitable lipids for formulating paliperidone-loaded NLCs. 

Solid lipids like glycerol monostearate, Precirol ATO 5 (glyceryl palmitostearate), Compritol 888 ATO (glyceryl behenate), Dynasan 114 (glycerol trimyristate), and stearic acid and liquid lipids like oleic acid, Labrafil^®^ M 2125 CS (linoleoyl polyoxyl-6 glycerides), Labrafil^®^ M 1944 CS (oleoyl polyoxyl-6 glycerides), and Labrafac^®^ CC (caprylic/capric triglycerides) were chosen due to their extensive utilization in the formulation of lipid nanocarriers [30,44]. The solubility of paliperidone in various lipids is presented in Table 2. Paliperidone showed the maximum solubility in glyceryl monostearate (29.83 µg/mg, solid lipid) and oleic acid (2.23 µg/mg, liquid lipid) among the lipids tested. Therefore, these two lipids were chosen for the formulation of NLCs. 

Additional ingredients like non-ionic stabilizers are generally included in NLC preparations to improve physical stability. Tween 80 has demonstrated greater potential to stabilize colloidal systems by steric effect, high drug loading, and good cell survival, hence suggesting safety [15,24]. Moreover, it was disclosed that the use of Tween 80 can form uniform assemblies with small particles caused by a decrease in interfacial tension between the dispersion and lipids used, thus making them suitable for drug delivery purposes [45]. The solubility of paliperidone in Tween 80 was ~46 µg/mg (Table 2).

### 3.2. Preliminary Batches of NLCs

Drugs entrapped in nanocarriers can improve pharmacological efficacy and reduce toxicity. Thus, EE is one of the most crucial components in the development of nanoparticulate formulations including NLCs. Nevertheless, the stability of the developed NLCs mainly depends on the type and amount of solid as well as liquid lipids, while the surfactants can also influence the EE [46]. To investigate the influence of the ratio of solid and liquid lipids and surfactants on the EE % of the drug, different formulations (PB1–PB9) were prepared with a total of 10% lipid content as per Table 3. Preliminary batches of NLCs (PB1–PB13) were prepared using the hot homogenization method by varying amounts of selected solid and liquid lipids (while the total content of lipids was fixed as 10% *w*/*w*), as well as the surfactant (Tween 80). The miscibility of the solid and liquid lipids used in formulating various batches (PB1–PB13) was found to be good in the current investigation. The sonication time was fixed at 15 min as the emulsion was relatively viscous and this duration was used in another study [47]. The amount of paliperidone was fixed (1%, *w*/*w*) and was below the saturation solubility of the drug in the ratio of solid and liquid lipids tested. Table 3 shows the results of the EE of prepared NLCs. The effect of the solid and liquid lipid ratio on the EE was evaluated by preparing PB1–PB9 batches, while the surfactant concentration was fixed at 2% based on the literature [34]. The results in Table 3 indicate considerable variation in the EE with a change in the amount of solid and liquid lipids. The EE increased from 75% to 88% in batches PB1–PB3, when the % of glycerol monostearate was decreased from 9% to 7% and the oleic acid (liquid lipid) amount was increased from 1% to 3%. The possible explanation for this observation is that an excessive amount of oleic acid may lead to the destruction of the lipid matrix, resulting in drug leakage and a subsequent decrease in drug EE as reported elsewhere [48]. The highest EE was observed in PB3 (88.45%) when a combination of solid lipid and liquid lipid ratio of 7:3 was used, suggesting the optimal EE requires a specific ratio of solid lipids. In batches PB4–PB9, wherein the amount of glycerol monostearate in total lipid was decreased and the oleic acid content was increased, a reduction in the EE value was observed (Table 3). To assess the effect of the amount of Tween 80 on EE, additional batches (PB10–PB13) were prepared where the surfactant quantity was varied between 0.5% and 4%. The data in Table 3 suggest the amount of Tween 80 considerably influences the EE, and the optimum level could be 2% (*w*/*w*), which shows the highest EE value. It was observed that when the Tween concentration was increased from 0.5% to 2%, there was a significant improvement in the EE, which may be due to the potential of Tween to disturb the crystal order and create more space to accommodate a greater amount of drug molecules in the matrix as mentioned elsewhere [49]. However, increasing the Tween concentration from 2% to 4% resulted in a decrease in EE, possibly due to lipid matrix saturation causing the drug to be expelled from the matrix [50].

### 3.3. Box-Behnken Design for Optimization of NLCs

Based on the results of initial trials, the ratio of solid–liquid lipids (X_1_; 7:3, 7.5:2.5, and 8:2) and concentrations of surfactant (X_2_; Tween 80: 1, 2, and 3%) were considered as independent variables. Indeed, the significant impact of sonication time on the EE of NLCs was described in the literature [33,51]. Hence, the ultrasonication time (X_3_) was selected as the third independent variable. The effect of selected variables on major formulation characteristics of NLCs including particle size (Y_1_), EE (Y_2_), and drug release (Y_3_) was evaluated. The Box–Behnken experimental design was used for the optimization of the proposed NLCs, with the selected factors at three different levels. The actual values of factors and the responses of various formulations studied (NL1-NL17) are summarized in Table 4. The ANOVA analysis of the data (Tables S2–S4) shows a significant impact of the selected factors on the dependent variables, which is further supported by the response surface plot and the quadratic equation’s coefficients (Y_1_, Y_2_, and Y_3_). The positive and negative signs of the regression equation’s terms signify increases and decreases in the response to the variables, respectively.

#### 3.3.1. Effect on Particle Size

One of the major physicochemical attributes that could affect drug delivery is the hydrodynamic diameter of NLCs. The observed size of prepared NLC particles ranged from 130.53 nm to 491.87 nm (Table 4), suggesting the selected factors influence the response Y_1_ (particle size). The software-generated linear equation with both quadratic and interaction terms proposed for the particle size based on the data analysis is as follows:

$$Y_{1}=+179.58+41.49X_{1}-42.50X_{2}+0.05X_{3}+51.33X_{1}X_{2}-18.26X_{1}X_{3}-14.00X_{2}X_{3}+126.48X_{1}^{2}+107.46X_{2}^{2}+48.76X_{3}^{2}$$

**Figure 1:**
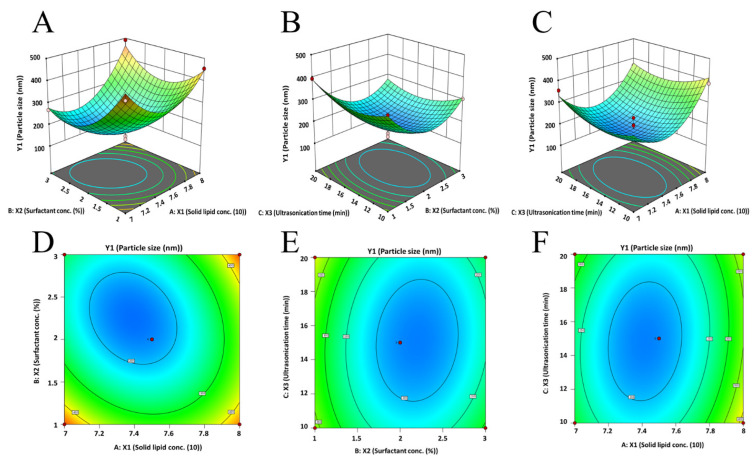
Three-dimensional surface response (**A**–**C**) and contour plot (**D**–**F**) showing the effect of three dependent variables on response (particle size, Y_1_).

**Figure 2:**
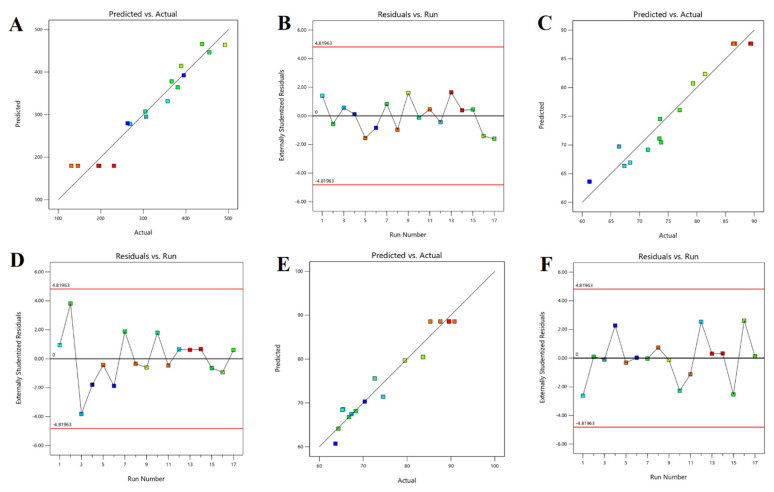
Predicted versus actual plots representing the linear correlation (**A**,**C**,**E**) and associated residual plots (**B**,**D**,**F**) for three responses studied.

The regression equation suggests that Y_1_ (particle size) had a negative relation (*p* < 0.05) with the concentration of Tween 80 (X_2_) while it had a positive relation (*p* < 0.05) with the ratio of solid and liquid lipids (X_1_). On the other hand, the duration of ultrasonication (X_3_) did not appear to have a noticeable effect (*p* > 0.05) on the particle size. The observed model F-value (13.28) in Table S2 implies this model is significant. The graph (Figure 1A,C) demonstrates that the particle size was reduced by a change from level −1 to 0 when altering the solid/liquid lipid ratio (X_1_). However, the particle size increased considerably when the ratio was further increased from 0 to +1. This reaction could be the consequence of the initial increase in solid lipid content that provides the optimum equilibrium between solid and liquid lipids, leading to NLCs with the smallest size [50]. On the other hand, when solid lipid concentration rises, more solid content accumulates, thereby increasing particle size (Figure 1A,C). Meanwhile, an increase in surfactant concentration (X_2_) first caused a significant drop in particle size, followed by a modest increase with a subsequent enhancement in surfactant concentration (Figure 1A,B). As described in the literature [52], an initial increase in surfactant concentration can decrease interfacial tension and create steric hindrance on the NLC surface, thereby promoting stability and preventing individual particles from aggregating. However, particle size was not significantly impacted by ultrasonication time (X_3_). With increasing ultrasonication duration, only minor changes were observed, as shown in Figure 1B,C, and the center point value was where the minimal particle size was discovered. The 3D image (Figure 1A–C) and 2D contour plots (Figure 1D–F) make it obvious how the particle size is impacted by the interplay of components X_1_, X_2,_ and X_3_. The greatest levels of X_1_ and X_2_ were associated with the largest particle size, whereas the center value of all the independent variables was associated with the smallest particle size. Figure 2A quantitatively compares the actual particle size values that were obtained with the predicted values (Table S5), whereas Figure 2B shows the corresponding residual plots for particle size.

#### 3.3.2. Effect on EE

The EE of NLCs is influenced by a combination of factors, including lipid composition, drug properties, drug-to-lipid ratio, manufacturing process, surface charge, particle size, drug loading method, and physicochemical conditions. Optimizing these parameters during NLC formulation can lead to an improved EE. The polynomial equation proposed for the EE is as follows:

$$Y_{2}=+87.64+3.62X_{1}-0.48X_{2}-3.26X_{3}+4.38X_{1}X_{2}-1.87X_{1}X_{3}+0.49X_{2}X_{3}-3.86X_{1}^{2}-8.95X_{2}^{2}-11.82X_{3}^{2}$$

**Figure 3:**
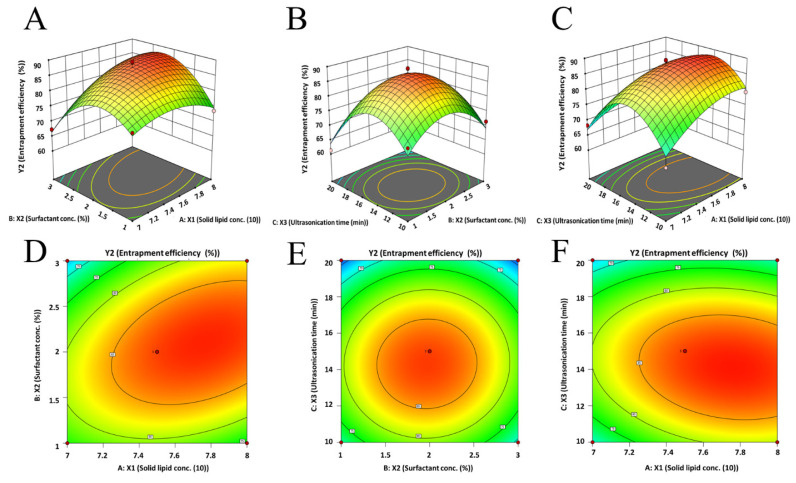
Three-dimensional surface response (**A**–**C**) and contour plot (**D**–**F**) showing the effect of three dependent variables on response (EE, Y_2_).

Table 4 shows that the EE of the prepared NLCs was within the range of 61.24% to 89.45%. The observed model F value (17.23) in Table S3 indicates the model is significant. The above quadratic equation signifies that the solid and liquid lipid ratio (X_1_) has a positive relation (increases the EE of paliperidone in prepared NLCs). The 3D response plot (Figure 3) indicates that the increase in solid/liquid lipid ratio from 7:3 to 7.5:2.5 leads to improvement in EE, while a further increase (8:2) leads to a minor decline in the EE value. One possible explanation for this behavior is that an initial increase in the amount of solid lipid may enhance drug solubility, resulting in a higher EE. However, a further increase in the lipid ratio led to a reduction in the amount of liquid lipid present, which in turn decreased the amount of drug that could be embedded in a liquid compartment and aided in its ejection from the solid matrix as observed earlier [53]. Similarly, the initial increase in surfactant concentration improved the EE value, while a higher concentration of Tween 80 led to a decrease in EE (Figure 3). The possible explanation for this observation is that an initial increase in surfactant concentration may improve paliperidone partitioning and make it easier for it to dissolve in both the aqueous (water with 1–3% Tween 80) and lipid phases. With a further increase in surfactant, the lipid matrix may become saturated, and a decrease in EE was shown as a result of the drug being expelled from the lipid matrix, which is described elsewhere [50]. In the case of ultrasonication, an initial increase in high-intensity motion could facilitate the transfer of drug particles from the aqueous (water with 1–3% Tween 80) solution to the lipid matrix, leading to good drug entrapment. However, prolonging the sonication period may result in the expulsion of loosely bound drugs from the matrix. Figure 2C presents a quantitative comparison of the actual and predicted values for EE (Table S5), while Figure 2D displays the corresponding residual plots for EE.

#### 3.3.3. Effect on Drug Release

The drug dissolution rate from NLC is generally influenced by formulation parameters like the type of lipids, quantity of surfactant, amount of active, and preparation method, in addition to the characteristics of the vesicle [54]. The amount of the drug released in 6 h from the prepared NLCs varied from 63.68% to 90.79% (Table 4). In addition, an initial burst release (2 h, 22–48%) followed by a sustained release (up to 6 h) was noticed with various NLCs. The polynomial expression suggested for the % drug release is depicted as follows:

$$Y_{3}=+88.56+3.18X_{1}+4.60X_{2}-2.84X_{3}+0.97X_{1}X_{2}-3.32X_{1}X_{3}+0.20X_{2}X_{3}-7.41X_{1}^{2}-10.19X_{2}^{2}-10.01X_{3}^{2}$$

**Figure 4:**
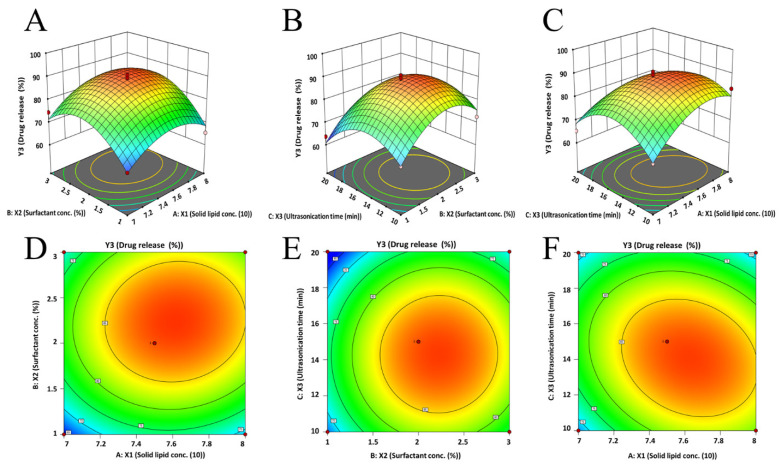
Three-dimensional surface response (**A**–**C**) and contour plot (**D**–**F**) showing the effect of three dependent variables on response (drug release, Y_3_).

This equation indicates that all of the formulation variables significantly influence the paliperidone release (6 h) from the drug-loaded NLCs. The observed model F-value (16.15) in Table S4 indicates the model is significant. The coefficient values of the different variables revealed that the ratio of solid and liquid lipids and surfactant had a positive relation (increases the drug release); however, the duration of ultrasonication had a negative relation (decreases the drug release from the prepared NLCs). The particular reasons for the effect of various factors studied can be explained using the quadratic equation generated by the software. As the ratio of lipids increased, the 3D response surface plot (Figure 4A,C) and 2D contour plot (Figure 4D,F) showed an initial increase and subsequent reduction in paliperidone release from NLCs. Similarly, there was an initial increase in drug release when the surfactant concentration (1% to 2%) and ultrasonication time (10 min to 15 min) were increased. However, a further increase in surfactant concentration (2% to 3%) and ultrasonication time (15 min to 20 min) decreases the drug release (Figure 4A–F). Figure 2E presents a quantitative comparison between the actual values and the predicted values for drug release, whereas Figure 2F depicts the corresponding residual plots for drug release.

#### 3.3.4. Optimization and Point Prediction

**Figure 5:**
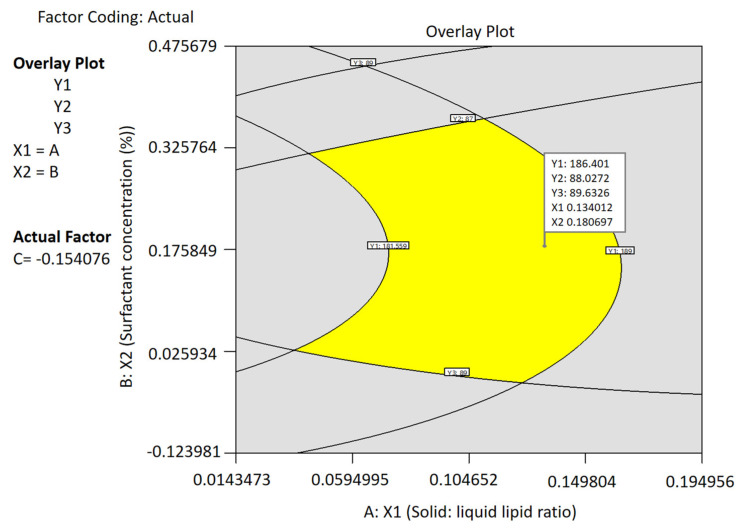
Overlay plot of the optimized NLC batch using design space.

By using the numerical point prediction optimization technique of the Design Expert program, the best formulation of the paliperidone NLC was chosen based on the criteria of achieving fair values of particle size, EE, and drug release. The formulation composition with a solid–liquid lipid ratio of 7.567:2.433, surfactant concentration of 2.18%, and ultrasonication time of 14.23 min was found to fulfill the requirements for an optimized paliperidone NLC formulation. Figure 5 displays the overlay plot of the optimized NLCs, while Table 5 presents the predicted and observed values. The findings indicate that the observed values of the responses aligned with the expected values, and the disparity between the estimated and observed values was negligible (<1%). As a result, it may be concluded that the mathematical solution obtained is reliable for forecasting all three responses tested (particle size, EE, and drug release) and the design model is mathematically valid. 

### 3.4. Characterization of NLCs

#### 3.4.1. Particle Characterization

**Figure 6:**
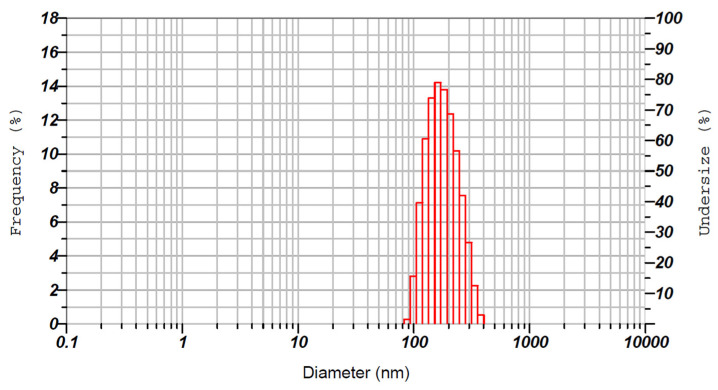
Representative image showing the particle size distribution of optimized NLC batch.

The physical characteristics of NLCs are important for assessing the mechanical behaviors they possess. The particle size of NLCs is of utmost importance as it influences various factors including, stability, drug release, and permeation across biological barriers [33]. In general, smaller particles with a narrow size distribution are less prone to aggregation and physical instability during storage. However, the particle size and distribution of NLCs can be significantly influenced by various factors such as the manufacturing process, as well as the quantities and types of lipids, surfactants, and drugs used in the formulation [33]. The uniformity of particles in the product is referred to as PDI, and a value range of 0.2–0.5 implies homogeneous dispersion and higher stability [55]. Hence, the average hydrodynamic size and PDI of the optimized NLCs were determined and were found to be ~186.3 nm and ~0.310, respectively (Figure 6). These values signify that the prepared NLCs are nanosized and have a narrow size distribution. Indeed, the low size of the prepared NLCs could be suitable for buccal therapy as it can easily diffuse through the buccal mucosa and provide rapid action [56]. In addition, the drug loading in the optimized NLCs was 8.2 ± 0.47%. 

#### 3.4.2. Zeta Potential

**Figure 7:**
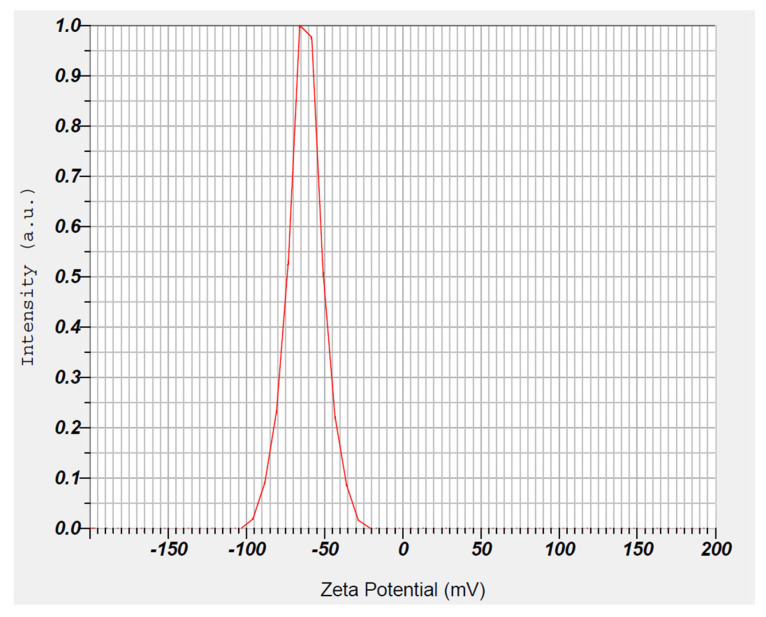
Representative image showing the zeta potential of optimized NLC batch.

The zeta potential represents the surface charge of nanocarriers and is a critical parameter that provides information on the electrophoretic mobility with surrounding dispersion. A higher surface charge is essential for a particle’s long-term stability because it creates a repulsive energy barrier that prevents vesicle aggregation [57]. The measured zeta potential of the optimized formulation showed a higher negative value (−62.3 mV), as seen in Figure 7. The observed negative surface charge could be attributed to the presence of free fatty acids contained in the solid and liquid lipids, which was described in the literature before [24]. The value observed here confirms the capability of the surface charges of the prepared NLCs to prevent vesicle agglomeration and hence lead to the adequate stability of the dispersion in SSF. Furthermore, the zeta potential of the developed carrier system contributes to mucosal delivery. Particles with negative charges have been shown to increase penetration through the negatively charged mucus by minimizing entrapment, whereas positively charged particles cause electrostatic interaction and promote cellular uptake [58].

#### 3.4.3. Morphology

**Figure 8:**
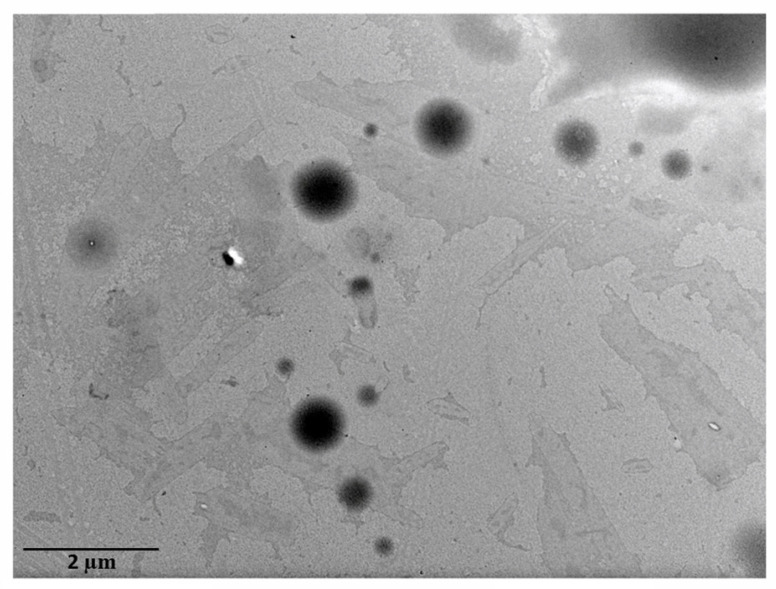
Transmission electron microscopy of optimized NLCs.

The external characteristics of nanocarriers have an impact on several physicochemical properties, including absorption, pharmacokinetics, and biodistribution [59]. The surface morphology of the prepared NLCs was studied using TEM to assess their size and shape. The TEM photomicrograph (Figure 8) portrays that paliperidone NLCs were spherical in shape, nanometer in size, smooth in morphology, nonadherent, and evenly distributed, with no aggregation of particles.

### 3.5. Characterization of NLC-Loaded Buccal Mucoadhesive Film

The film composition consists of a combination of HPMC and PC and was selected based on their favorable biological and mucoadhesive properties suitable for buccal films reported earlier [17,27,28]. The embodiments used in formulating film have HPMC (2% *w*/*v*) and PC (0.5% *w*/*v*) polymers and glycerol (1% *w*/*v*). The concentrations of HPMC and PC were selected based on earlier studies [23]. Optimized NLCs were loaded during the film preparation and evaluated for all major characteristics. The prepared NLC-loaded buccal film had light weight (55 ± 1.33 mg, 1cm^2^), low thickness (1.09 ± 0.06 mm), adequate drug content (1.95 ± 0.12 mg/cm^2^), good content uniformity (97.34 ± 5.75%), a pH close to saliva (6.41 ± 0.03), and the absence of any visual indications of cuts or openings. The flexibility of the film was tested to evaluate its ability to bend and avoid breaking during application. The results of the folding endurance test revealed that the prepared buccal film had an average score of 309, which is well within the ideal range [60]. Overall, these results were comparable to those in the reported literature and signify that the film is suitable for buccal application and is not likely to irritate the buccal mucosa [61]. 

**Figure 9:**
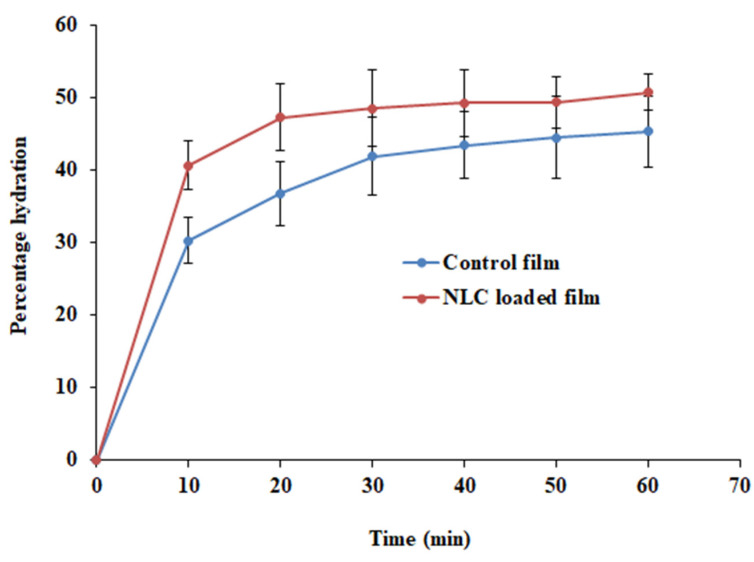
Percentage hydration of NLC-loaded and control (plain-drug-loaded) films.

The swelling of a polymeric film is a critical parameter that plays a crucial role in the adhesion of the formulation to the biological membrane. Polymeric swelling results in the disentanglement and relaxation of polymer chains, which in turn facilitates the penetration of the mucous membrane during bioadhesion [23]. Hence, the % hydration of the NLC-loaded film and plain-drug-loaded film (control) was determined and is depicted in Figure 9. It was noticed that the % hydration was rapid (10 min), and the values were ~40% and ~30% with NLC-loaded and control films, respectively. This rapid hydration is possible due to the greater water uptake in the initial period by the hydrophilic polymer (HPMC). The hydration further increased and reached equilibrium in 20–30 min, signifying the establishment of hydrogen bonds between the two polymers (HPMC and PC) [15]. The profile also shows that the hydration of NLC-loaded films was greater (*p* < 0.001) than that of the control film.

Tensile strength refers to the highest stress applied to the region of the film at which the film tears. This is a mechanical property measured to ensure the strength of films and is relatively low with soft and weak polymers. The tensile strength values of NLC-loaded (0.407 N/cm^2^) and placebo (0.394 N/cm^2^) films were comparable and indicated sufficient strength [37].

Mucoadhesion plays a crucial role in the effectiveness of buccal therapy, as inadequate bonding to the mucosa can result in the dislodging of the film from the site of application. The mucoadhesive strength of both PA-Film (7.0 ± 0.4 N) and PA-NLC film (7.8 ± 0.5 N) indicates that the films exhibit sufficient bioadhesive strength, which is attributed to the combination of mucoadhesive polymers PC and HPMC [62]. 

### 3.6. Degree of Crystallinity

**Figure 10:**
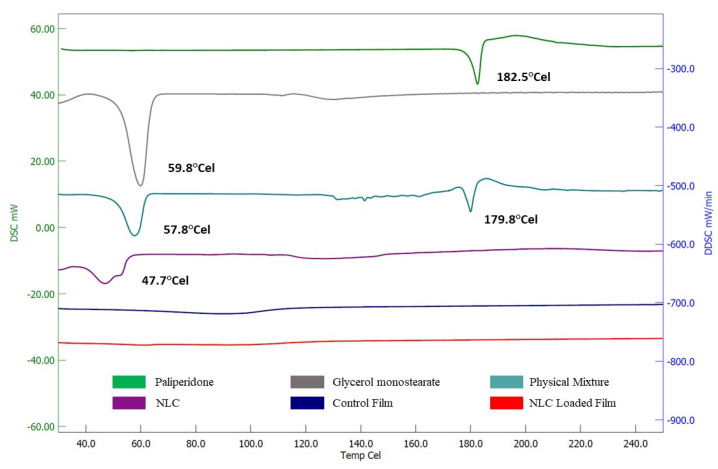
DSC image of paliperidone, glycerol monostearate, physical mixture, NLC, control film (plain-drug-loaded film), and optimized NLC-loaded film.

For thermal analysis, a DSC experiment was conducted to assess the melting and crystalline state of the formulation embodiments in pure form as well as in composition [63]. The DSC spectra of pure paliperidone, glycerol monostearate (solid lipid), the physical mixture, NLCs, the control film (plain-drug-loaded film), and the optimized NLC-loaded film are presented in Figure 10. The DSC profile showed a single discernible melting endothermic peak for pure paliperidone (182.5 °C) and glycerol monostearate (59.8 °C). The physical mixture exhibited two endothermic peaks at temperatures of 57.8 °C and 179.8 °C, corresponding to glycerol monostearate and the drug, respectively. The NLC formulation showed a weak peak of glycerol monostearate which was shifted towards a lower temperature (47.7 °C), which indicates the solid lipid is dissolved in liquid lipid (oleic acid) and the crystalline nature of lipids is lowered as described in the literature [24,64]. It has also been reported that the crystalline nature of lipids decreases due to the interaction between solid and liquid lipids in the NLCs; in addition, the lipids exist in an oily state inside the NLCs and disorganize the crystalline order [63]. Notably, there was no paliperidone endotherm in the DSC spectra of the control film or NLC-loaded film. The complete disappearance of the characteristic endothermic melting peak of paliperidone in both films proved that the drug was either converted into an amorphous form (completely dissolved) or molecularly dispersed in the lipid matrix.

### 3.7. Spectral Analysis

**Figure 11:**
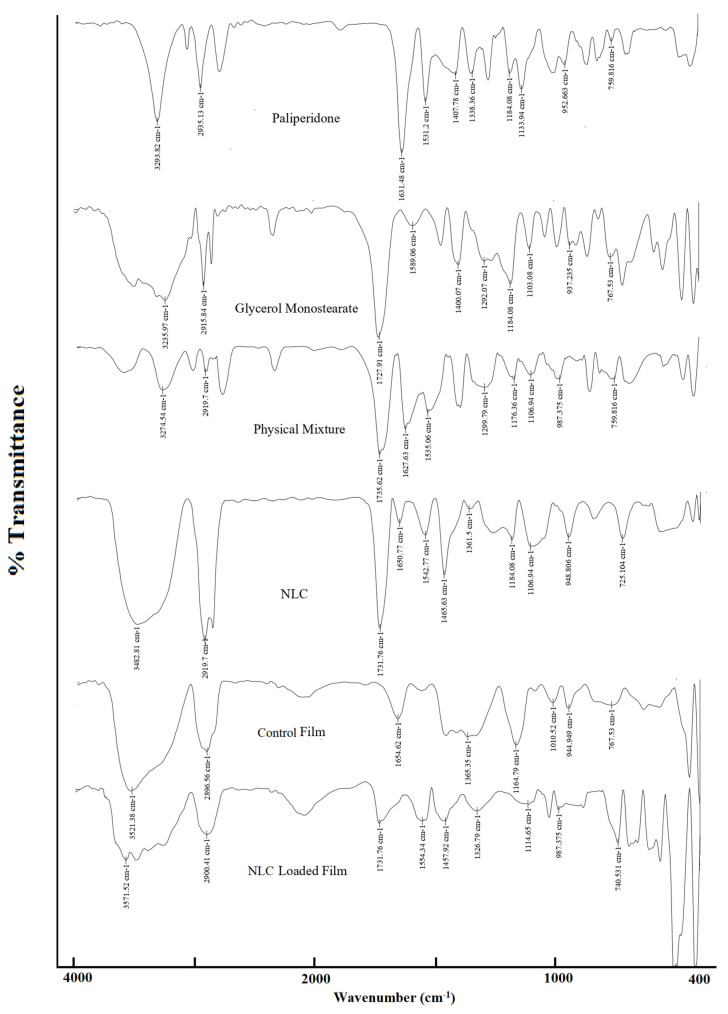
FTIR spectra of paliperidone, glycerol monostearate, physical mixture, NLC, control film (plain-drug-loaded film), and optimized NLC-loaded film.

FTIR spectroscopy was used to identify the functional groups of paliperidone and glycerol monostearate, and the peaks for the formulations were compared to assess the possible drug–excipient interactions. The spectra of the drug show all the characteristic peaks of paliperidone like 3293 cm^−1^ (-OH stretching of alcohol), 2935 cm^−1^ (-CH stretching of alkane), 1531 cm^−1^ (-NO stretching of nitro), 1407 cm^−1^ (-OH bonding of alcohol), 1338 cm^−1^ (-CF stretching of fluoro), 1184 cm^−1^ (-CN stretching), 1133 cm^−1^ (-CO stretching), 952 cm^−1^ (C=C bending), and 759 cm^−1^ (-CH bending) (Figure 11). The FTIR spectrum of the physical mixture exhibits the characteristic and distinct peaks of both paliperidone and glycerol monostearate, indicating the absence of any interaction between paliperidone and the excipients. Similarly, the control film showed all major peaks corresponding to paliperidone. The optimized NLCs and NLC-loaded film demonstrated the existence of the paliperidone spectral bands with a minor decrease in intensity, probably due to the successful encapsulation of the drug inside NLCs [65]. The absence of any additional peaks or major shifts in drug peak wavelength in the optimized NLC-loaded film confirms the absence of any interactions of paliperidone with the embodiments used in the fabrication of the NLCs and film.

### 3.8. Drug Release from Films

**Figure 12:**
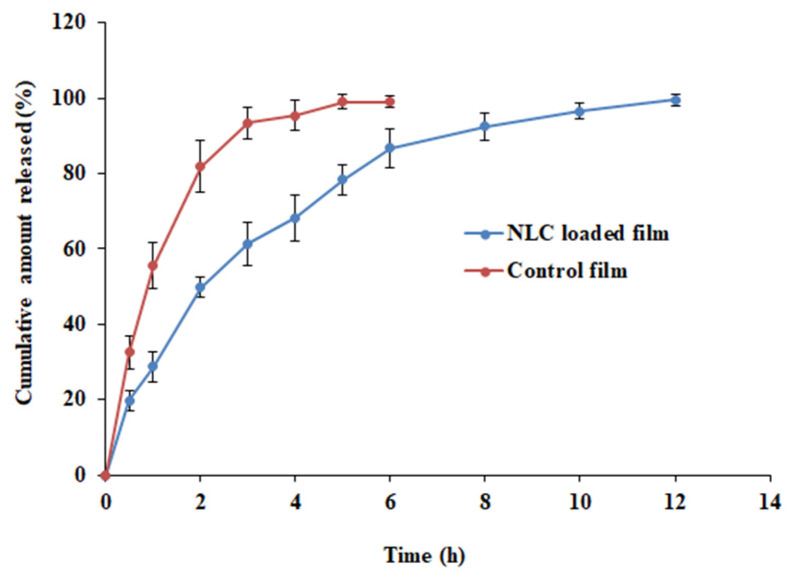
Drug release profiles of NLC-loaded and control (plain-drug-loaded) films.

In general, mucoadhesive formulations can adhere to buccal tissues and thereby provide a long residence time. Lipid nanoparticles provide a high surface area and can encapsulate hydrophobic drugs like PA, improving their solubility and stability within the buccal film matrix. The lipid nanoparticles within the buccal film matrix can modulate the release of the drug by different mechanisms. By incorporating lipid nanoparticles into buccal films, the release of PA can be tuned to achieve sustained and controlled drug release. Hence, evaluation of drug release from the formulation is essential to understand the performance in vivo. The release profiles of paliperidone NLC-loaded film and control film are shown in Figure 12. The paliperidone release from NLC-loaded films was very different (*p* < 0.0005) from the control. The release seemed prolonged and steady for 12 h when the drug was encapsulated in a carrier system, while a biphasic pattern (~80% in 2 h) with the complete release was noticed in plain-drug-loaded film. This observation demonstrated the importance of developing a mucoadhesive film for the buccal administration of NLCs. In the case of nanocarrier-loaded films, the release of the drug occurs due to the detachment of particles from the polymer matrix and then diffusion through the matrix. Additional factors involved in the drug release are water diffusion, resistance exerted by the lipid core, partition coefficient, swelling, and film erosion. Thus, the sustained release kinetics of paliperidone observed here could be linked to the above factors as observed by other researchers [54,66,67]. Furthermore, the extended release pattern observed here is beneficial for buccal therapy as the nanocarriers detached from the buccal film are likely to diffuse into the mucosal epithelium and eventually release the drug to provide a sustained effect. The release kinetics of the PA-NLC film was found to follow the Weibull model kinetics with a higher r^2^ value (0.9909), low SSR (73.6053), and Fischer ratio (8.1784). In addition, the observed n value (0.4472) was lesser than 0.75, indicating Fickian diffusion [68]. It is also reported that Weibull model drug release phenomena are generally exhibited by swellable polymeric nanoparticles [69].

### 3.9. Drug Permeation

**Figure 13:**
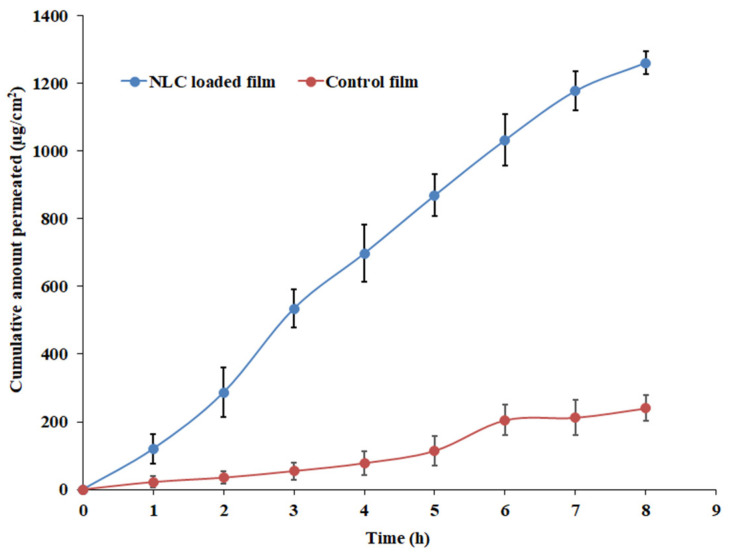
Ex vivo permeation profiles of NLC-loaded and control (plain-drug-loaded) films.

Ex vivo permeation studies are a decisive element for assessing the absorption kinetics of drugs/carriers and can be useful in predicting in vivo behavior [14]. In general, both the physiological characteristics of the biological membrane and the properties of the active compound have a significant impact on the permeation of the diffusant across the membrane. Moreover, lipid nanoparticles within the mucoadhesive polymer would allow it to adhere to the mucosal surfaces in the oral cavity. This increased adhesion prolongs the residence time, facilitating prolonged drug release, better drug absorption, and permeation through the buccal mucosa. The lipid nanoparticles may also interact with the mucus layer, promoting drug permeation across the mucosal barrier. The permeation profiles of paliperidone NLC-loaded film and control film through the excised rabbit mucosa are shown in Figure 13. Significantly higher drug permeation (*p* < 0.005) was noticed for the NLC-loaded film than for the control film. The NLC-loaded film profile indicates that permeation of paliperidone was rapid from the first hour itself (lag time 0.68 h) and steadily increased (flux value, ~167.33 µg/cm^2^/h) with time. The cumulative amount of drug permeated in 8 h was ~1260.56 µg/cm^2^, while the permeability coefficient was ~8.37 × 10^−2^ cm/h. Overall, the flux with the NLC-loaded buccal film was ~5 times higher than that with the control film. The higher permeation noticed here indicates that the NLCs are capable of easily detaching from the film and quickly diffusing through the buccal mucosa. Indeed, the nature of NLC vesicles, occlusive effect, particle size, and surfactant all together could accelerate the passive transport of drugs through the buccal cell membrane as explained in various studies [47,70]. On the other hand, the control film showed relatively slow and low permeation (flux, 32.29 µg/cm^2^/h). The data observed here indicated the superiority of the NLC-loaded film over the plain-drug-containing film. 

### 3.10. In Vivo Study

**Figure 14:**
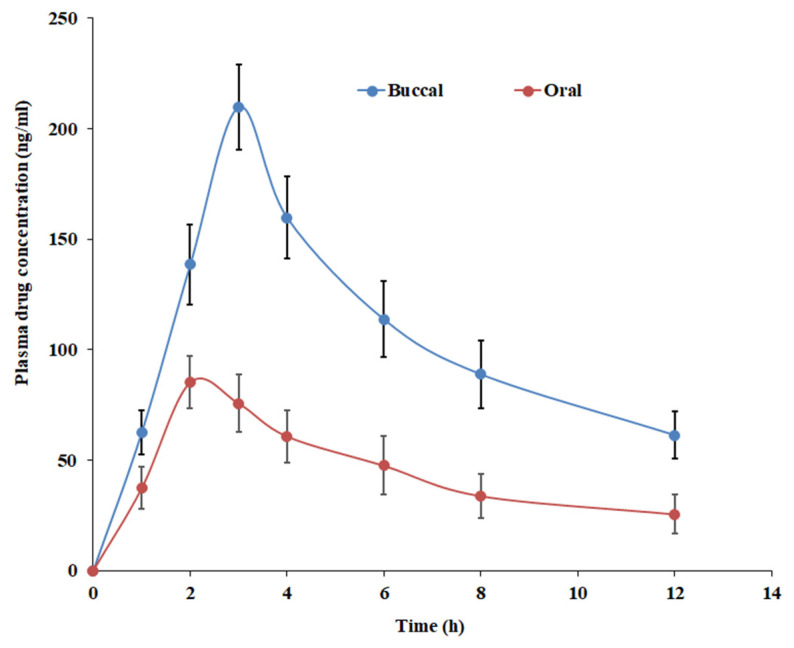
In vivo pharmacokinetics profiles of paliperidone from oral suspension and NLC-loaded film.

Animal experiments are commonly used to gain a better understanding of the parameters that influence the effective absorption of drugs from developed films. The in vivo absorption of paliperidone from the developed buccal film and the oral suspension (control) was compared by administering 2 mg of the drug. The plasma concentration profiles and the results of various pharmacokinetic parameters tested are presented in Figure 14 and Table 6, respectively. The paliperidone plasma profiles of buccal and oral therapy were found to be different (*p* < 0.01), suggesting that the route of administration affects the drug’s pharmacokinetics. The drug absorption seemed rapid in the buccal route, with ~62 ng/mL seen in 1 h. The plasma drug concentration continued to increase and reached the T_max_ in 3 h in buccal administration, while it was early in oral therapy (2 h). Indeed, a significant difference in C_max_ values (*p* < 0.0001) and absorption (AUC_0–12_, *p* < 0.0001) was seen between two different routes, with a considerably higher amount being noticed with the paliperidone NLC-loaded film as compared to control suspension. It was demonstrated that the paliperidone NLC-loaded buccal film has a much higher bioavailability (236% relative bioavailability) than the control, with appreciable changes in plasma peak exposure. These findings suggest that the NLC-loaded film not only promotes long-term retention of paliperidone but also enhances its bioavailability, both of which may be advantageous for once-daily regimen therapy.

## 4. Conclusions

Lipid nanoparticles offer advantages such as improved drug solubility, stability, controlled release mechanisms, enhanced mucoadhesion, permeation enhancement, and potential for targeted drug delivery. These factors contribute to the overall effectiveness of buccal films loaded with lipid nanoparticles in regulating the release of PA and improving its therapeutic outcomes. This study showcases the design and development of a paliperidone-loaded NLC buccal film, which holds promise as an alternative treatment option for schizophrenia. The hot homogenization method and film casting technique were used to prepare NLCs and films, respectively. The development of the paliperidone-loaded NLC buccal film was performed by selecting appropriate lipids and preparing ideal NLCs by optimizing formulation variables affecting particle characteristics by employing the Box-Behnken design. The optimized NLC formulation was successfully embedded in a polymeric film containing HPMC and PC. The physicochemical properties of the NLC-loaded buccal mucoadhesive film were found to be ideal for buccal application. FTIR and DSC analysis data demonstrated the compatibility of the drug and additives in the formulation. The sustained drug release and higher flux (~5-fold higher than the control) seen with the film indicate the potential of the developed film. Pharmacokinetic data in rabbits demonstrated a larger C_max_ (*p* < 0.0001), an improvement in AUC_0–12_ (*p* < 0.0001), and better relative bioavailability (236%) as compared to the control suspension. The promising evidence presented here suggests that the developed NLC-loaded buccal film has immense potential for efficiently managing schizophrenia and other schizoaffective or delusional diseases. 

## Figures and Tables

**Table 1:** Actual values used for experimental runs.

Factors	Actual (Coded) Values		
	Low (−1)	Medium (0)	High (+1)
Independent variables			
X_1_ = Solid–liquid lipid ratio	7:3	7.5:2.5	8:2
X_2_ = Surfactant concentration (%)	1	2	3
X_3_ = Ultrasonication time (min)	10	15	20
Dependent variable	Targets		
Y_1_ = Particle size (nm)	Minimum		
Y_2_ = EE %	Maximum		
Y_3_ = Drug release (%)	Maximum		

EE, entrapment efficiency.

**Table 2:** Solubility of paliperidone in various lipids and surfactants tested.

Lipid/Surfactant	Amount of Paliperidone Dissolved (µg/mg)
Glycerol monostearate	29.83 ± 0.98
Precirol ATO 5	15.78 ± 0.67
Compritol 888 ATO	19.44 ± 1.06
Dynasan 114	20.11 ± 0.74
Stearic acid	14.24 ± 0.85
Oleic acid	2.23 ± 0.64
Labrafil^®^ M 2125 CS	0.94 ± 0.53
Labrafil^®^ M 1944 CS	1.67 ± 0.38
Labrafac^®^ CC	1.45 ± 0.68
Tween 80	45.58 ± 1.72

The values represent the average of three trials.

**Table 3:** Composition of different batches of NLCs prepared for assessing the excipient’s effect on EE % of paliperidone.

Batches	Composition			Entrapment Efficiency (%)
	Paliperidone (%, *w*/*w*)	Glycerol Monostearate: Oleic Acid Ratio	Tween 80 (%, *w*/*w*)	
PB1	1	9:1	2	75.43
PB2	1	8:2	2	80.23
PB3	1	7:3	2	88.45
PB4	1	6:4	2	80.65
PB5	1	5:5	2	75.73
PB6	1	4:6	2	70.12
PB7	1	3:7	2	63.56
PB8	1	2:8	2	55.98
PB9	1	1:9	2	45.30
PB10	1	7:3	0.5	58.46
PB11	1	7:3	1	75.33
PB12	1	7:3	3	68.93
PB13	1	7:3	4	63.47

**Table 4:** The actual values and corresponding responses observed for design batches.

Formulation	Actual Values			Response Values			PDI	Zeta Potential (mV)
	Solid–Liquid Lipid Ratio, X_1_	Surfactant Concentration (%), X_2_	Ultrasonication Time (min), X_3_	Particle Size (nm), Y_1_	Entrapment Efficiency (%), Y_2_	Drug Release (%), Y_3_		
NL1	7:3	2	20	357.24 ± 43.66	68.34 ± 3.28	65.25 ± 3.35	0.65	−54.6
NL2	8:2	2	20	366.56 ± 38.24	73.75 ± 3.95	68.32 ± 4.08	0.75	−48.2
NL3	7:3	2	10	306.57 ± 29.88	66.42 ± 3.50	67.31 ± 3.82	0.39	−56.2
NL4	7.5:2.5	1	20	394.78 ± 47.61	61.29 ± 3.08	63.68 ± 5.04	0.44	−52.4
NL5	7.5:2.5	2	15	130.53 ± 27.32	86.42 ± 4.22	87.58 ± 4.66	0.37	−49.2
NL6	7.5:2.5	3	20	262.91 ± 30.64	61.24 ± 3.34	70.37 ± 4.12	0.57	−37.8
NL7	7.5:2.5	1	10	380.71 ± 35.82	73.48 ± 3.94	66.73 ± 3.06	0.51	−53.6
NL8	7.5:2.5	2	15	146.08 ± 25.68	86.65 ± 4.71	90.79 ± 4.28	0.48	−66.3
NL9	8:2	3	15	491.87 ± 44.20	81.43 ± 4.30	79.48 ± 4.37	0.69	−45.7
NL10	7.5:2.5	3	10	304.83 ± 37.08	71.47 ± 2.97	72.62 ± 4.16	0.45	−31.4
NL11	7.5:2.5	2	15	196.45 ± 33.18	86.35 ± 4.15	85.32 ± 4.72	0.26	−41.4
NL12	7:3	1	15	269.07 ± 39.27	67.34 ± 3.01	74.54 ± 3.97	0.45	−53.1
NL13	7.5:2.5	2	15	230.69 ± 28.16	89.32 ± 4.64	89.52 ± 4.60	0.51	−43.8
NL14	7.5:2.5	2	15	194.15 ± 30.42	89.45 ± 4.28	89.57 ± 4.49	0.20	−53.3
NL15	8:2	1	15	455.32 ± 35.61	73.56 ± 3.65	65.43 ± 3.88	0.59	−34.7
NL16	8:2	2	10	388.94 ± 39.37	79.31 ± 3.44	83.64 ± 4.14	0.67	−39.5
NL17	7:3	1	15	437.86 ± 48.94	76.99 ± 3.90	64.36 ± 3.90	0.54	−40.6

**Table 5:** Estimated and observed values of optimized NLC formulation.

Y_1_ (Particle Size) nm		Y_2_ (EE) %		Y_3_ (Drug Release) %	
Estimated	Observed	Estimated	Observed	Estimated	Observed
186.40	186.33	88.03	88.18	89.63	89.19

EE, entrapment efficiency; nm, nanometer.

**Table 6:** Pharmacokinetic data of paliperidone in plasma for oral and buccal therapy in rabbits. Data are the average of six animals.

Parameter	Paliperidone NLC-Loaded Film (Mean ± SD)	Paliperidone Suspension (Mean ± SD)
C_max_ (ng/mL)	209.75 ± 19.27 *	85.87 ± 11.87
T_max_ (h)	3	2
AUC_0–12_ (ng. h/mL)	1266.94 ± 103.66 *	536.17 ± 60.33
Relative bioavailability (%)	236	100

T_max_, time of maximum concentration; C_max_, maximum concentration; AUC, area under the plasma concentration–time curve; NLCs, nanostructured lipid carriers. * *p* < 0.0001 vs. paliperidone suspension.

## Data Availability

The data presented in this study are contained within the article.

## Supplementary Materials

The following supporting information can be downloaded at: https://www.mdpi.com/article/10.3390/pharmaceutics15112530/s1, Table S1. Concentrations of various factors used for optimization; Table S2. ANOVA for quadratic model for particle size; Table S3. ANOVA for quadratic model for entrapment efficiency; Table S4. ANOVA for quadratic model for drug release; Table S5. Predicted values for various dependent variables.

## References

[1] Lobo M.C., Whitehurst T.S., Kaar S.J., Howes O.D. (2022). New and emerging treatments for schizophrenia: A narrative review of their pharmacology, efficacy and side effect profile relative to established antipsychotics. Neurosci. Biobehav. Rev..

[2] Fellner C. (2017). New Schizophrenia Treatments Address Unmet Clinical Needs. P T Peer-Rev. J. Formul. Manag..

[3] Devrimci-Ozguven H., Atmaca M., Baran Z., Cengisiz C., Çinar C., Erol A., Genç Y., Karadağ H., Karakülah K., Karasu U. (2019). Efficacy and Safety of Paliperidone Palmitate Treatment in Patients with Schizophrenia: A Real-World Multicenter, Retrospective, Mirror-Image Study. J. Clin. Psychopharmacol..

[4] Rehman S., Nabi B., Javed A., Khan T., Iqubal A., Ansari M.J., Baboota S., Ali J. (2022). Unraveling enhanced brain delivery of paliperidone-loaded lipid nanoconstructs: Pharmacokinetic, behavioral, biochemical, and histological aspects. Drug Deliv..

[5] Mauri M.C., Reggiori A., Paletta S., Di Pace C., Altamura A.C. (2017). Paliperidone for the treatment of schizophrenia and schizoaffective disorders—A drug safety evaluation. Expert Opin. Drug Saf..

[6] Wesołowska A., Jastrzębska-Więsek M., Cios A., Partyka A. (2020). The preclinical discovery and development of paliperidone for the treatment of schizophrenia. Expert Opin. Drug Discov..

[7] Mali S., Oza N. (2022). Formulation and optimization of Paliperidone palmitate biodegradable injectable microspheres using Box-Behnken design. J. Drug Deliv. Sci. Technol..

[8] Rehman S., Nabi B., Baboota S., Ali J. (2021). Tailoring lipid nanoconstructs for the oral delivery of paliperidone: Formulation, optimization and in vitro evaluation. Chem. Phys. Lipids.

[9] Patel M.R., Patel R.B., Bhatt K.K., Patel B.G., Gaikwad R.V. (2016). Paliperidone microemulsion for nose-to-brain targeted drug delivery system: Pharmacodynamic and pharmacokinetic evaluation. Drug Deliv..

[10] Thimmasetty J., Ghosh T., Nayak N.S., Raheem A. (2021). Oral bioavailability enhancement of paliperidone by the use of cocrystalization and precipitation inhibition. J. Pharm. Innov..

[11] Jee J.P., Kim Y.H., Lee J.H., Min K.A., Jang D.J., Jin S.G., Cho K.H. (2023). Paliperidone-Cation Exchange Resin Complexes of Different Particle Sizes for Controlled Release. Pharmaceutics.

[12] Raval S., Jani P., Patil P., Thakkar P., Sawant K. (2021). Enhancement of bioavailability through transdermal drug delivery of paliperidone palmitate-loaded nanostructured lipid carriers. Ther. Deliv..

[13] Deruyver L., Rigaut C., Gomez-Perez A., Lambert P., Haut B., Goole J. (2023). In vitro Evaluation of Paliperidone Palmitate Loaded Cubosomes Effective for Nasal-to-Brain Delivery. Int. J. Nanomed..

[14] Jacob S., Nair A.B., Boddu S.H.S., Gorain B., Sreeharsha N., Shah J. (2021). An updated overview of the emerging role of patch and film-based buccal delivery systems. Pharmaceutics.

[15] Tzanova M.M., Hagesaether E., Tho I. (2021). Solid lipid nanoparticle-loaded mucoadhesive buccal films—Critical quality attributes and in vitro safety & efficacy. Int. J. Pharm..

[16] Montero-Padilla S., Velaga S., Morales J.O. (2017). Buccal Dosage Forms: General Considerations for Pediatric Patients. AAPS PharmSciTech.

[17] Shipp L., Liu F., Kerai-Varsani L., Okwuosa T.C. (2022). Buccal films: A review of therapeutic opportunities, formulations & relevant evaluation approaches. J. Control. Release Off. J. Control. Release Soc..

[18] Al-Dhubiab B.E., Nair A.B., Kumria R., Attimarad M., Harsha S. (2016). Development and evaluation of buccal films impregnated with selegiline-loaded nanospheres. Drug Deliv..

[19] Al-Dhubiab B.E., Nair A.B., Kumria R., Attimarad M., Harsha S. (2015). Formulation and evaluation of nano based drug delivery system for the buccal delivery of acyclovir. Colloids Surf. B Biointerfaces.

[20] Beloqui A., Solinís M., Rodríguez-Gascón A., Almeida A.J., Préat V. (2016). Nanostructured lipid carriers: Promising drug delivery systems for future clinics. Nanomed. Nanotechnol. Biol. Med..

[21] Fonseca-Santos B., Silva P.B., Rigon R.B., Sato M.R., Chorilli M. (2020). Formulating SLN and NLC as Innovative Drug Delivery Systems for Non-Invasive Routes of Drug Administration. Curr. Med. Chem..

[22] Basahih T.S., Alamoudi A.A., El-Say K.M., Alhakamy N.A., Ahmed O.A.A. (2020). Improved Transmucosal Delivery of Glimepiride via Unidirectional Release Buccal Film Loaded with Vitamin E TPGS-Based Nanocarrier. Dose-Response A Publ. Int. Hormesis Soc..

[23] Kraisit P., Limmatvapirat S., Luangtana-Anan M., Sriamornsak P. (2018). Buccal administration of mucoadhesive blend films saturated with propranolol loaded nanoparticles. Asian J. Pharm. Sci..

[24] Tetyczka C., Griesbacher M., Absenger-Novak M., Fröhlich E., Roblegg E. (2017). Development of nanostructured lipid carriers for intraoral delivery of Domperidone. Int. J. Pharm..

[25] Kamboj S., Bala S., Nair A.B. (2010). Solid lipid nanoparticles: An effective lipid based technology for poorly water soluble drugs. Int. J. Pharm. Sci. Rev. Res..

[26] Van N.H., Vy N.T., Van Toi V., Dao A.H., Lee B.-J. (2022). Nanostructured lipid carriers and their potential applications for versatile drug delivery via oral administration. OpenNano.

[27] Zhu Z., Zhai Y., Zhang N., Leng D., Ding P. (2013). The development of polycarbophil as a bioadhesive material in pharmacy. Asian J. Pharm. Sci..

[28] Fonseca-Santos B., Chorilli M. (2018). An overview of polymeric dosage forms in buccal drug delivery: State of art, design of formulations and their in vivo performance evaluation. Mater. Sci. Eng. C Mater. Biol. Appl..

[29] Rudragangaiah S., Bhatta R.G., Kotappa S.B.B. (2019). Stability-Indicating RP-HPLC Method for the Quantification of Paliperidone in Bulk and Solid Dosage Form to Establish Validation and Stability Indicating Parameters. Order.

[30] Mahmood A., Rapalli V.K., Gorantla S., Waghule T., Singhvi G. (2022). Dermatokinetic assessment of luliconazole-loaded nanostructured lipid carriers (NLCs) for topical delivery: QbD-driven design, optimization, and in vitro and ex vivo evaluations. Drug Deliv. Transl. Res..

[31] Gomaa E., Fathi H.A., Eissa N.G., Elsabahy M. (2022). Methods for preparation of nanostructured lipid carriers. Methods.

[32] Wu K.W., Sweeney C., Dudhipala N., Lakhani P., Chaurasiya N.D., Tekwani B.L., Majumdar S. (2021). Primaquine Loaded Solid Lipid Nanoparticles (SLN), Nanostructured Lipid Carriers (NLC), and Nanoemulsion (NE): Effect of Lipid Matrix and Surfactant on Drug Entrapment, in vitro Release, and ex vivo Hemolysis. AAPS PharmSciTech.

[33] Subramaniam B., Siddik Z.H., Nagoor N.H. (2020). Optimization of nanostructured lipid carriers: Understanding the types, designs, and parameters in the process of formulations. J. Nanoparticle Res..

[34] Wang W., Chen L., Huang X., Shao A. (2017). Preparation and Characterization of Minoxidil Loaded Nanostructured Lipid Carriers. AAPS PharmSciTech.

[35] Shete M.B., Deshpande A.S., Shende P. (2023). Enhancement of in-vitro anti-oral cancer activities of silymarin using dispersion of nanostructured lipid carrier in mucoadhesive in-situ gel. Int. J. Pharm..

[36] Nair A.B., Al-Dhubiab B.E., Shah J., Vimal P., Attimarad M., Harsha S. (2018). Development and evaluation of palonosetron loaded mucoadhesive buccal films. J. Drug Deliv. Sci. Technol..

[37] Ammar H.O., Ghorab M.M., Felton L.A., Gad S., Fouly A.A. (2016). Effect of Antiadherents on the Physical and Drug Release Properties of Acrylic Polymeric Films. AAPS PharmSciTech.

[38] Nair A.B., Al-Dhubiab B.E., Shah J., Jacob S., Saraiya V., Attimarad M., SreeHarsha N., Akrawi S.H., Shehata T.M. (2020). Mucoadhesive buccal film of almotriptan improved therapeutic delivery in rabbit model. Saudi Pharm. J..

[39] Jug M., Hafner A., Lovrić J., Kregar M.L., Pepić I., Vanić Ž., Cetina-Čižmek B., Filipović-Grčić J. (2018). An overview of in vitro dissolution/release methods for novel mucosal drug delivery systems. J. Pharm. Biomed. Anal..

[40] Nair A., Vyas H., Shah J., Kumar A. (2011). Effect of permeation enhancers on the iontophoretic transport of metoprolol tartrate and the drug retention in skin. Drug Deliv..

[41] Anroop B., Ghosh B., Parcha V., Kumar A., Khanam J. (2005). Synthesis and comparative skin permeability of atenolol and propranolol esters. J. Drug Deliv. Sci. Technol..

[42] Jacob S., Nair A.B., Morsy M.A. (2022). Dose conversion between animals and humans: A practical solution. Indian J. Pharm. Educ. Res..

[43] Persson L.C., Porter C.J., Charman W.N., Bergström C.A. (2013). Computational prediction of drug solubility in lipid based formulation excipients. Pharm. Res..

[44] Marathe S., Shadambikar G., Mehraj T., Sulochana S.P., Dudhipala N., Majumdar S. (2022). Development of α-Tocopherol Succinate-Based Nanostructured Lipid Carriers for Delivery of Paclitaxel. Pharmaceutics.

[45] Izza N., Suga K., Okamoto Y., Watanabe N., Bui T.T., Wibisono Y., Fadila C.R., Umakoshi H. (2021). Systematic Characterization of Nanostructured Lipid Carriers from Cetyl Palmitate/Caprylic Triglyceride/Tween 80 Mixtures in an Aqueous Environment. Langmuir ACS J. Surf. Colloids.

[46] Cirri M., Maestrini L., Maestrelli F., Mennini N., Mura P., Ghelardini C., Di Cesare Mannelli L. (2018). Design, characterization and in vivo evaluation of nanostructured lipid carriers (NLC) as a new drug delivery system for hydrochlorothiazide oral administration in pediatric therapy. Drug Deliv..

[47] Kraisit P., Sarisuta N. (2018). Development of Triamcinolone Acetonide-Loaded Nanostructured Lipid Carriers (NLCs) for Buccal Drug Delivery Using the Box-Behnken Design. Molecules.

[48] Zhang K., Lv S., Li X., Feng Y., Li X., Liu L., Li S., Li Y. (2013). Preparation, characterization, and in vivo pharmacokinetics of nanostructured lipid carriers loaded with oleanolic acid and gentiopicrin. Int. J. Nanomed..

[49] Elmowafy M., Shalaby K., Ali H.M., Alruwaili N.K., Salama A., Ibrahim M.F., Akl M.A., Ahmed T.A. (2019). Impact of nanostructured lipid carriers on dapsone delivery to the skin: In vitro and in vivo studies. Int. J. Pharm..

[50] Thapa C., Ahad A., Aqil M., Imam S.S., Sultana Y. (2018). Formulation and optimization of nanostructured lipid carriers to enhance oral bioavailability of telmisartan using Box–Behnken design. J. Drug Deliv. Sci. Technol..

[51] Javed S., Mangla B., Almoshari Y., Sultan M.H., Ahsan W. (2022). Nanostructured lipid carrier system: A compendium of their formulation development approaches, optimization strategies by quality by design, and recent applications in drug delivery. Nanotechnol. Rev..

[52] Agrawal M., Saraf S., Pradhan M., Patel R.J., Singhvi G., Ajazuddin, Alexander A. (2021). Design and optimization of curcumin loaded nano lipid carrier system using Box-Behnken design. Biomed. Pharmacother. Biomed. Pharmacother..

[53] Qadir A., Aqil M., Ali A., Warsi M.H., Mujeeb M., Ahmad F.J., Ahmad S., Beg S. (2020). Nanostructured lipidic carriers for dual drug delivery in the management of psoriasis: Systematic optimization, dermatokinetic and preclinical evaluation. J. Drug Deliv. Sci. Technol..

[54] Gordillo-Galeano A., Mora-Huertas C.E. (2018). Solid lipid nanoparticles and nanostructured lipid carriers: A review emphasizing on particle structure and drug release. Eur. J. Pharm. Biopharm. Off. J. Arbeitsgemeinschaft Fur Pharm. Verfahrenstechnik e.V.

[55] Sneha K., Kumar A. (2022). Nanoemulsions: Techniques for the preparation and the recent advances in their food applications. Innov. Food Sci. Emerg. Technol..

[56] Khosa A., Reddi S., Saha R.N. (2018). Nanostructured lipid carriers for site-specific drug delivery. Biomed. Pharmacother. Biomed. Pharmacother..

[57] Gordillo-Galeano A., Mora-Huertas C.E. (2021). Hydrodynamic diameter and zeta potential of nanostructured lipid carriers: Emphasizing some parameters for correct measurements. Colloids Surf. A Physicochem. Eng. Asp..

[58] Veider F., Akkuş-Dağdeviren Z.B., Knoll P., Bernkop-Schnürch A. (2022). Design of nanostructured lipid carriers and solid lipid nanoparticles for enhanced cellular uptake. Int. J. Pharm..

[59] Varela-Fernández R., García-Otero X., Díaz-Tomé V., Regueiro U., López-López M., González-Barcia M., Isabel Lema M., Javier Otero-Espinar F. (2022). Lactoferrin-loaded nanostructured lipid carriers (NLCs) as a new formulation for optimized ocular drug delivery. Eur. J. Pharm. Biopharm. Off. J. Arbeitsgemeinschaft Fur Pharm. Verfahrenstechnik e.V.

[60] Kumria R., Nair A.B., Al-Dhubiab B.E. (2014). Loratidine buccal films for allergic rhinitis: Development and evaluation. Drug Dev. Ind. Pharm..

[61] Okafor N.I., Ngoepe M., Noundou X.S., Krause R.W.M. (2019). Nano-enabled liposomal mucoadhesive films for enhanced efavirenz buccal drug delivery. J. Drug Deliv. Sci. Technol..

[62] Kraisit P., Limmatvapirat S., Nunthanid J., Sriamornsak P., Luangtana-Anan M. (2017). Preparation and Characterization of Hydroxypropyl Methylcellulose/Polycarbophil Mucoadhesive Blend Films Using a Mixture Design Approach. Chem. Pharm. Bull..

[63] Elkomy M.H., Elmowafy M., Shalaby K., Azmy A.F., Ahmad N., Zafar A., Eid H.M. (2021). Development and machine-learning optimization of mucoadhesive nanostructured lipid carriers loaded with fluconazole for treatment of oral candidiasis. Drug Dev. Ind. Pharm..

[64] Ho H.N., Le H.H., Le T.G., Duong T.H.A., Ngo V.Q.T., Dang C.T., Nguyen V.M., Tran T.H., Nguyen C.N. (2022). Formulation and characterization of hydroxyethyl cellulose-based gel containing metronidazole-loaded solid lipid nanoparticles for buccal mucosal drug delivery. Int. J. Biol. Macromol..

[65] Zewail M.B., Asaad G.F., Swellam S.M., Abd-Allah S.M., Hosny S.K., Sallah S.K., Eissa J.E., Mohamed S.S., El-Dakroury W.A. (2022). Design, characterization and in vivo performance of solid lipid nanoparticles (SLNs)-loaded mucoadhesive buccal tablets for efficient delivery of Lornoxicam in experimental inflammation. Int. J. Pharm..

[66] Na Y.G., Huh H.W., Kim M.K., Byeon J.J., Han M.G., Lee H.K., Cho C.W. (2020). Development and evaluation of a film-forming system hybridized with econazole-loaded nanostructured lipid carriers for enhanced antifungal activity against dermatophytes. Acta Biomater..

[67] Arunprasert K., Pornpitchanarong C., Piemvuthi C., Siraprapapornsakul S., Sripeangchan S., Lertsrimongkol O., Opanasopit P., Patrojanasophon P. (2022). Nanostructured lipid carrier-embedded polyacrylic acid transdermal patches for improved transdermal delivery of capsaicin. Eur. J. Pharm. Sci. Off. J. Eur. Fed. Pharm. Sci..

[68] Corsaro C., Neri G., Mezzasalma A.M., Fazio E. (2021). Weibull Modeling of Controlled Drug Release from Ag-PMA Nanosystems. Polymers.

[69] Azadi S., Ashrafi H., Azadi A. (2017). Mathematical modeling of drug release from swellable polymeric nanoparticles. J. Appl. Pharm. Sci..

[70] Hosny K.M., Sindi A.M., Ali S., Alharbi W.S., Hajjaj M.S., Bukhary H.A., Badr M.Y., Mushtaq R.Y., Murshid S.S.A., Almehmady A.M. (2022). Development, optimization, and evaluation of a nanostructured lipid carrier of sesame oil loaded with miconazole for the treatment of oral candidiasis. Drug Deliv..

