# Supplementary material for: Design, Development, Evaluation, and In Vivo Performance of Buccal Films Embedded with Paliperidone-Loaded Nanostructured Lipid Carriers
Source: Pharmaceutics. 2023 Oct 25;15(11):2530. doi: 10.3390/pharmaceutics15112530 (PMC10674218; doi:10.3390/pharmaceutics15112530)
Supplement: Supplementary file 1 [file pharmaceutics-15-02530-s001.zip › pharmaceutics-2630682-supplementary.pdf]

pharmaceutics

Design, Development, Evaluation and In Vivo Performance of Buccal Films Embedded with Paliperidone Loaded Nanostructured Lipid Carriers

Table S1. Concentrations of various factors used for optimization.

Formulation	Actual values of factors		
	Solid: liquid lipid ratio, X_1	Surfactant concentration (%), X_2	Ultrasonication time (min), X_3
NL1	7:3	2	20
NL2	8:2	2	20
NL3	7:3	2	10
NL4	7.5:2.5	1	20
NL5	7.5:2.5	2	15
NL6	7.5:2.5	3	20
NL7	7.5:2.5	1	10
NL8	7.5:2.5	2	15
NL9	8:2	3	15
NL10	7.5:2.5	3	10
NL11	7.5:2.5	2	15
NL12	7:3	3	15
NL13	7.5:2.5	2	15
NL14	7.5:2.5	2	15
NL15	8:2	1	15
NL16	8:2	2	10
NL17	7:3	1	15
Optimized formulation	7.57:2.43	2.18	14.23

Table S2. ANOVA for Quadratic model for particle size.

Source	Sum of Squares	df	Mean Square	F-value	p-value	
Model	1.794E+05	9	19932.00	13.28	0.0013	significant
A-X1	13773.85	1	13773.85	9.18	0.0191	
B-X2	14449.15	1	14449.15	9.63	0.0172	
C-X3	0.0242	1	0.0242	0.0000	0.9969	
AB	10541.13	1	10541.13	7.03	0.0329	
AC	1334.08	1	1334.08	0.8891	0.3771	
BC	783.72	1	783.72	0.5223	0.4933	
A^2	67361.92	1	67361.92	44.89	0.0003	
B^2	48626.22	1	48626.22	32.41	0.0007	
C^2	10011.71	1	10011.71	6.67	0.0363	
Residual	10503.08	7	1500.44			
Lack of Fit	3865.81	3	1288.60	0.7766	0.5650	not significant
Pure Error	6637.27	4	1659.32			
Cor Total	1.899E+05	16				

Table S3. ANOVA for Quadratic model for entrapment efficiency.

Source	Sum of Squares	df	Mean Square	F-value	p-value	
Model	1364.78	9	151.64	17.23	0.0006	significant
A-X1	104.84	1	104.84	11.91	0.0107	
B-X2	1.84	1	1.84	0.2094	0.6611	
C-X3	84.89	1	84.89	9.64	0.0172	
AB	76.74	1	76.74	8.72	0.0213	
AC	13.99	1	13.99	1.59	0.2479	
BC	0.9604	1	0.9604	0.1091	0.7508	
A^2	62.78	1	62.78	7.13	0.0320	
B^2	337.01	1	337.01	38.28	0.0005	
C^2	588.41	1	588.41	66.84	< 0.0001	
Residual	61.62	7	8.80			
Lack of Fit	51.39	3	17.13	6.70	0.0487	significant
Pure Error	10.23	4	2.56			
Cor Total	1426.41	16				

Table S4. ANOVA for Quadratic model for drug release.

Source	Sum of Squares	df	Mean Square	F-value	p-value	
Model	1578.07	9	175.34	16.15	0.0007	significant
A-X1	80.71	1	80.71	7.43	0.0295	
B-X2	169.37	1	169.37	15.60	0.0055	
C-X3	64.30	1	64.30	5.92	0.0452	
AB	3.74	1	3.74	0.3449	0.5755	
AC	43.96	1	43.96	4.05	0.0841	
BC	0.1600	1	0.1600	0.0147	0.9068	
A^2	231.30	1	231.30	21.31	0.0024	
B^2	437.35	1	437.35	40.29	0.0004	
C^2	422.25	1	422.25	38.90	0.0004	
Residual	75.99	7	10.86			
Lack of Fit	57.62	3	19.21	4.18	0.1003	not significant
Pure Error	18.37	4	4.59			
Cor Total	1654.06	16				

Table S5. Predicted values for various dependent variables.

Formulation	Dependent Variables		
	Particle size (nm), Y_1	Entrapment efficiency (%), Y_2	Drug release (%), Y_3
NL1	331.65	66.95	68.43
NL2	378.11	70.45	68.16
NL3	295.02	69.72	67.47
NL4	392.36	63.60	60.71
NL5	179.58	87.64	88.56
NL6	279.37	63.62	70.32
NL7	364.25	71.10	66.78
NL8	179.58	87.64	88.56
NL9	463.86	82.35	79.70
NL10	307.25	69.16	75.59
NL11	179.58	87.64	88.56
NL12	278.20	66.35	71.41
NL13	179.58	87.64	88.56
NL14	179.58	87.64	88.56
NL15	446.19	74.55	68.56
NL16	414.53	80.70	80.46
NL17	465.87	76.07	64.14
